# Serial femtosecond crystallography structure of cytochrome *c* oxidase at room temperature

**DOI:** 10.1038/s41598-017-04817-z

**Published:** 2017-07-03

**Authors:** Rebecka Andersson, Cecilia Safari, Robert Dods, Eriko Nango, Rie Tanaka, Ayumi Yamashita, Takanori Nakane, Kensuke Tono, Yasumasa Joti, Petra Båth, Elin Dunevall, Robert Bosman, Osamu Nureki, So Iwata, Richard Neutze, Gisela Brändén

**Affiliations:** 1 Department of Chemistry and Molecular Biology, University of Gothenburg, Box 462, SE-40530 Gothenburg, Sweden; 2 RIKEN Spring-8 Center, 1-1-1 Kouto, Sayo-cho, Sayo-gun, Hyogo, 679-5148 Japan; 3 Department of Cell Biology, Graduate School of Medicine, Kyoto University, Yoshidakonoe-cho, Sakyo-ku, Kyoto, 606-8501 Japan; 4 Department of Biological Sciences, Graduate School of Science, University of Tokyo, 2-11-16 Yayoi, Bunkyo-ku, Tokyo, 113-0032 Japan; 5 Japan Synchrotron Radiation Research Institute, 1-1-1 Kouto, Sayo-cho, Sayo-gun, Hyogo, 679-5198 Japan

## Abstract

Cytochrome *c* oxidase catalyses the reduction of molecular oxygen to water while the energy released in this process is used to pump protons across a biological membrane. Although an extremely well-studied biological system, the molecular mechanism of proton pumping by cytochrome *c* oxidase is still not understood. Here we report a method to produce large quantities of highly diffracting microcrystals of *ba*_3_-type cytochrome *c* oxidase from *Thermus thermophilus* suitable for serial femtosecond crystallography. The room-temperature structure of cytochrome *c* oxidase is solved to 2.3 Å resolution from data collected at an X-ray Free Electron Laser. We find overall agreement with earlier X-ray structures solved from diffraction data collected at cryogenic temperature. Previous structures solved from synchrotron radiation data, however, have shown conflicting results regarding the identity of the active-site ligand. Our room-temperature structure, which is free from the effects of radiation damage, reveals that a single-oxygen species in the form of a water molecule or hydroxide ion is bound in the active site. Structural differences between the *ba*_3_-type and *aa*_3_-type cytochrome *c* oxidases around the proton-loading site are also described.

## Introduction

Cytochrome *c* oxidases (C*c*Os) are the terminal enzymes of the respiratory chains in mitochondria and many bacteria. They are integral membrane bound complexes that catalyse the reduction of molecular oxygen to water and utilize the energy released to translocate protons across the cell membrane. The free energy stored in this electrochemical proton gradient is used by the cell to synthesize ATP and to drive transmembrane transport. The C*c*Os have been intensively studied over the last six decades. A vast body of spectroscopic work has detailed the time course of electron and proton movements within the enzymes. Cytochrome *c* delivers electrons from the positive side of the membrane at the same time as protons are taken up from the negative side of the membrane to the heme *a*_3_ - Cu_B_ active site, where the reaction with oxygen takes place. This creates a charge separation across the membrane which is further enhanced by the additional pumping of protons from the positive to the negative side of the membrane (see ref. 1 for a review).

In the *aa*_3_-type C*c*Os, including the mitochondrial enzyme, two functional proton pathways have been localized; the D- and the K-pathway. It is generally understood that the four protons to be pumped, as well as two substrate protons to be used in the chemical reaction, are taken up through the D-pathway and the remaining two substrate protons are taken up through the K-pathway, for each complete reaction cycle^2–5^. In the *ba*_3_-type C*c*O from the thermophilic bacterium *Thermus thermophilus* (*ba*_3_ C*c*O), a single proton pathway is used to transport all four substrate protons from the cytoplasmic (negative) side of the membrane to the active site, as well as the two protons to be translocated all the way across to the periplasmic (positive) side^6, 7^. Thus, the *ba*_3_ C*c*O pumps protons with a lower stoichiometry compared to the *aa*_3_-type C*c*Os, with only two (as opposed to four) protons pumped per reaction cycle^7^. Despite these differences, the general belief is that all members of the C*c*O superfamily utilize a common mechanism for proton pumping. The key question is; what is the structural mechanism linking oxygen reduction to proton pumping?

Many members of the C*c*O superfamily have been structurally characterized in detail^8–12^. In addition to the oxidized resting state of the enzyme there are structures available also of the reduced form with and without bound ligand^13, 14^, CO-bound form before and after flash-photolysis^15^ and different mutant forms of the enzyme^9, 16, 17^. All previous structures, however, have been solved using either frozen crystals, or at room temperature but under conditions likely to cause radiation damage^18, 19^. In addition, there are no high-resolutions structures of any of the intermediate states that occur during the reaction cycle and consequently there is no detailed structural understanding of how unidirectional proton translocation takes place.

X-ray Free Electron Lasers (XFELs) offer the possibility to collect diffraction data using extremely intense X-ray pulses of femtoseconds in duration, enabling diffraction to occur before the onset of radiation damage^20^. This avoidance of radiation damage is of particular importance when studying proteins that contain metal cofactors such as the C*c*Os^21, 22^. With the introduction of serial femtosecond crystallography (SFX)^23^, it has become possible to collect diffraction data on a continuous stream of micrometer sized crystals that flow across a focused XFEL beam. Each microcrystal gives rise to one diffraction image that is then merged with data collected from other microcrystals to recover a complete diffraction data set. Here we present the first room-temperature structure of a C*c*O determined by SFX. This opens up for future possibilities to track structural changes as oxygen is reduced to water and protons are pumped across the membrane in real time.

## Results

### Crystallization, characterization, LCP injection and data collection

**Figure 1:**
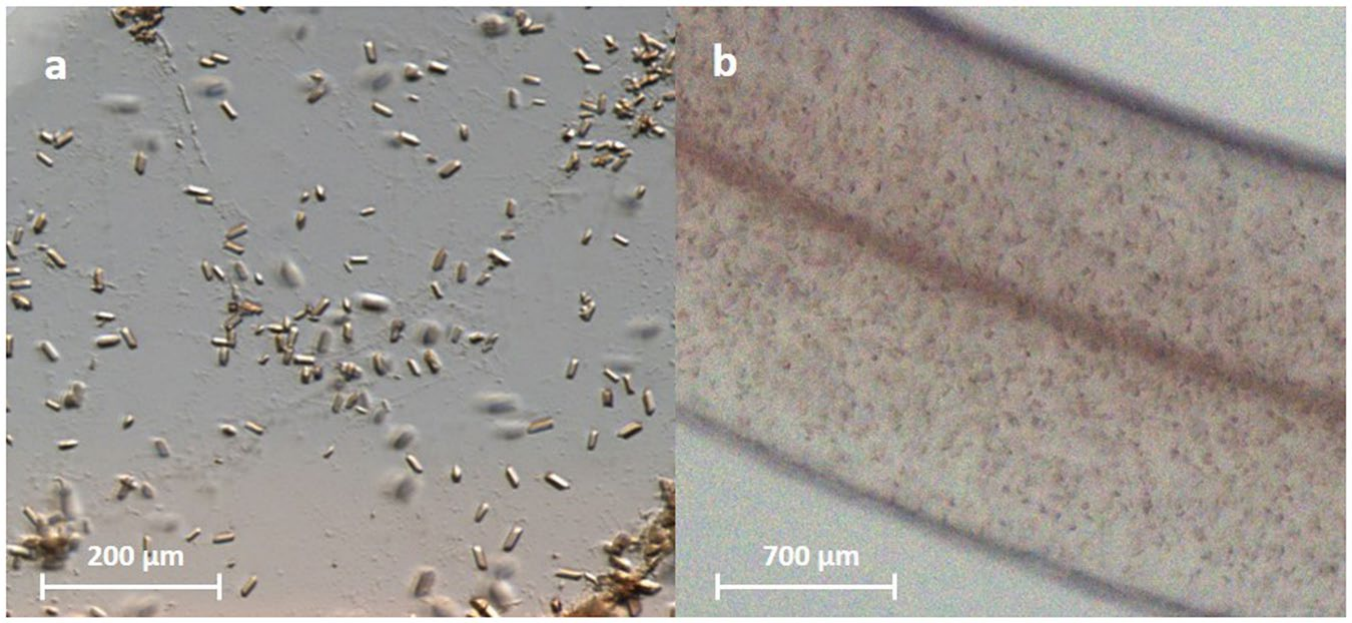
Microcrystals of *ba*_3_ C*c*O. (**a**) Microcrystals in an LCP drop. (**b**) Microcrystals in a string of LCP.

Conditions known to produce lipidic cubic phase (LCP) crystals of *ba*_3_ C*c*O from *Thermus thermophilus* sufficiently large for synchrotron based crystallography^24^ were modified to yield showers of micrometer sized crystals. A protocol was then developed to prepare microcrystals in glass plates or glass syringes by surrounding the protein-lipid mixture with precipitant solution, similar to a method previously described^25^. Under these conditions a large number of well-diffracting 5–20 µm sized *ba*_3_ C*c*O crystals appeared after 2–3 days (Fig. 1).

The oxidation state of *ba*_3_ C*c*O after crystallization was checked by recording an optical absorbance spectrum from microcrystals as prepared in the LCP phase (Supplementary Figure S1). The peak at 415 nm shows that the enzyme is in the oxidised state within the microcrystals. A spectrum was also recorded of microcrystals reduced by the addition of sodium dithionite (Supplementary Figure S1). This caused a shift of the absorption peak to 427 nm with a shoulder at 445 nm, and the appearance of peaks at 528 nm and 560 nm, characteristic of the reduced enzyme.

**Figure 2:**
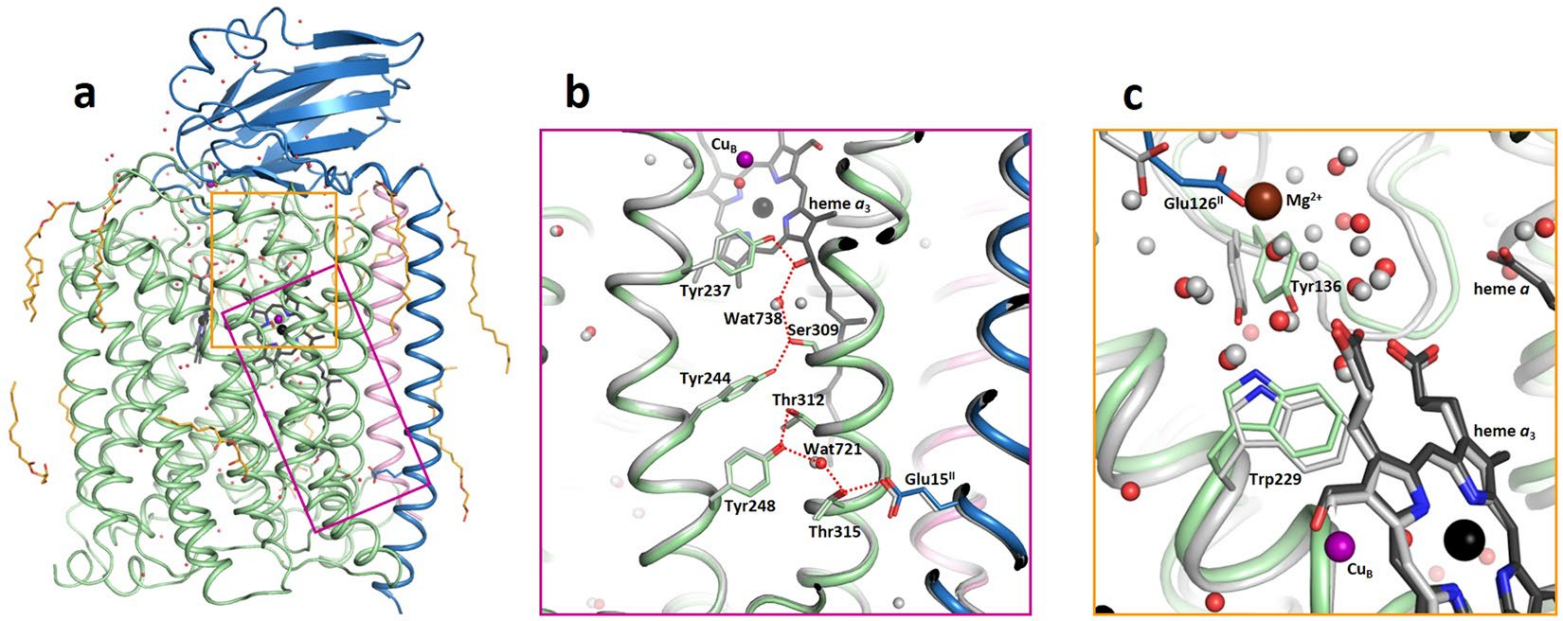
Room-temperature SFX structure of *ba*_3_ C*c*O. (**a**) Overall structure with subunit I in green, subunit II in blue, subunit IIa in pink, heme groups in black, copper ions in purple and lipids in orange. (**b**) Close-up view of the proton pathway where the SFX structure is shown in green/blue/pink and the cryo-LCP structure (PDB code 3S8G) is shown in grey. Water molecules are shown in red (SFX) and grey (cryo-LCP). (**c**) Close-up view of the area around the presumed proton-loading site. The SFX structure is shown in green/blue and the bovine heart *aa*_3_-type C*c*O (PDB code 3WG7) in grey. The magnesium ion of the bovine heart structure is shown in brown. Water molecules are shown in red (SFX) and grey (bovine heart). Heme *a*_3_ is shown in black (SFX) and grey (bovine heart). The amino acid numbering refers to the *ba*_3_ C*c*O.

X-ray diffraction data were collected at SPring-8 Angstrom Compact Free Electron Laser (SACLA) with highly brilliant X-ray pulses at 7.6 keV and approximately 10 fs in duration. Microcrystals were delivered into the X-ray focus at a flow rate of 0.48 µl/min using an LCP injector developed at SACLA^26^. A total of 87,057 frames were collected out of which 11,374 were identified as containing a diffraction pattern using the software Cheetah^27^, of which 8,211 frames were indexed and merged using CrystFEL^28^. Thus a complete diffraction data set was collected from approximately 50 minutes of data collection using 25 µl of sample. The structure was solved by molecular replacement using Phaser^29^ with PDB code 3S8F as a search model, and refined to a resolution of 2.3 Å. In the following text we refer to the room-temperature structure described here as the SFX structure (Fig. 2a).

### Room-temperature *ba*_3_ C*c*O structure

A number of structures of *ba*_3_ C*c*O are available in the protein data bank (PDB), both using vapor diffusion and LCP crystallization methods^11, 17, 24^. The highest resolution structures at 1.8 Å resolution were obtained by LCP crystallization (PDB codes 3S8F and 3S8G for wild type and A120F mutant enzyme, respectively)^24^. All previously reported structures, however, have been determined using crystals cooled to cryogenic temperatures (~100 K). In the following discussion we use the PDB code 3S8G structure for comparison (referred to as the cryo-LCP structure) as it has the best defined electron density.

Overall, the SFX structure (Fig. 2a) agrees very well with previously solved structures, with a C_α_ root mean-square deviation of 0.32 Å compared to the cryo-LCP structure. The space group, C2, is the same as the cryo-LCP structure with similar unit cell parameters. We can confirm the presence of 13 out of 20 lipid molecules modelled in the cryo-LCP structure, and we also localize two new lipid tails associated with subunit I (Supplementary Figure S2). In the SFX structure we resolve 92 water molecules, which compares with 225 observed in the cryo-LCP structure. This difference in number of modelled water molecules is possibly due to the lower resolution of the SFX structure, or because of increased disorder of water molecules when diffraction data are recorded at room temperature.

### Active site ligand

The active site of C*c*Os is made up of heme *a*_3_, Cu_B_ and surrounding residues. This site is where molecular oxygen binds in to the reduced heme *a*_3_ iron and the O=O double bond is cleaved through the uptake of four electrons and four protons, producing two water molecules. In several structures of oxidized C*c*O the electron density at the active site has been interpreted to indicate the presence of peroxide (O_2_^2−^) bound between the heme *a*_3_ iron and Cu_B_^24, 30, 31^. This interpretation has been questioned by Kaila and co-workers who claim that a stable peroxide in the active site of oxidized C*c*O is unlikely^32^. Their arguments were based upon quantum chemical calculations in combination with spectroscopic data and re-refinement of two X-ray structures. For the *ba*_3_ C*c*O, as many as 34 structures have been deposited in the PDB from crystals grown using the method of vapor diffusion^11^ and from lipidic cubic phase crystallization^24^. These structures are summarized in Supplementary Table S1. Structures resulting from vapor diffusion crystallization show conflicting results regarding the ligand bound in the active site, where the omit map electron density appears spherical in some cases (best fitted with a single-oxygen species such as water) and elongated in other cases (best fitted with a peroxide species) (Supplementary Figure S3). The authors attribute this uncertainty to effects of temperature as well as time of exposure to X-rays^24, 33^. All fourteen structures of *ba*_3_ C*c*O resulting from LCP crystallization display somewhat elongated omit map electron density in the active site, which has repeatedly been interpreted to indicate a bound peroxide molecule (Supplementary Table S1). However, this elongated density is potentially due to X-ray induced reduction^24^.

**Figure 3:**
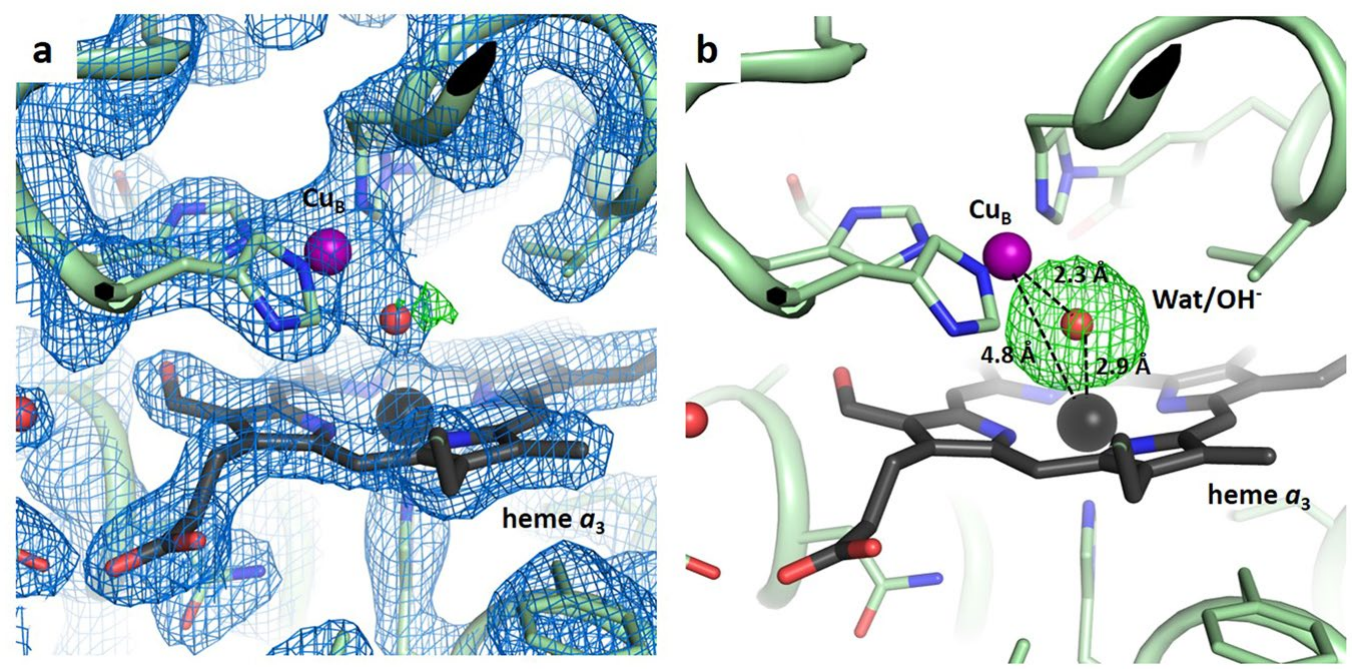
Active site structure of the *ba*_3_ C*c*O from SFX data. (**a**) View of the heme *a*_3_ – Cu_B_ active site with a water molecule or hydroxide ion bound. The 2F_o_-F_c_ density (blue) is contoured at 1.5 σ and the F_o_-F_c_ difference density (green) at 4.0 σ. (**b**) The F_o_-F_c_ omit map density, calculated without the water molecule, is contoured at 4.5 σ. Approximate distances between the heme *a*_3_ – Cu_B_, the heme *a*_3_ – ligand and Cu_B_ – ligand are indicated.

**Figure 4:**
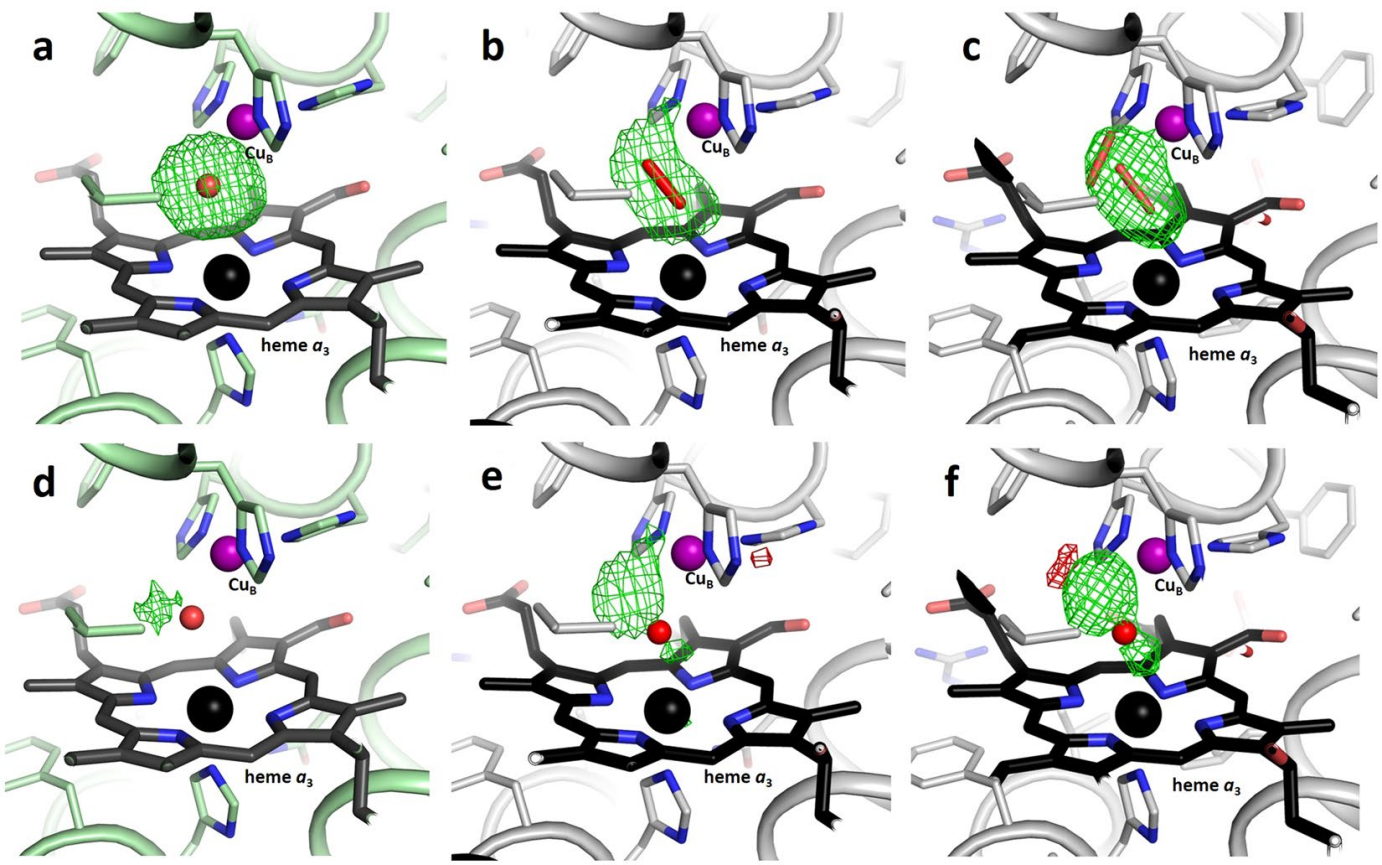
Comparison of active site electron densities. (**a**–**c**) F_o_-F_c_ omit map densities (green) contoured at 4.5 σ, calculated without any active site ligands. (**a**) The omit map density of *ba*_3_ C*c*O from SFX data. The bound water molecule/hydroxide ion is shown in red. (**b**) The omit map density of *ba*_3_ C*c*O from cryo-LCP data (PDB code 3S8G). The bound peroxide molecule is shown in red. (**c**) The omit map density of bovine heart *aa*_3_-type C*c*O from XFEL data of large crystals at cryo temperature (PDB code 3WG7). Two bound peroxide molecules with partial occupancies are shown in red. (**d**–**f**) F_o_-F_c_ difference densities calculated with a water molecule bound in the active site are shown in green (positive) and red (negative), contoured at +4.0/−4.0 σ. The bound water molecules are shown in red. (**d**) The F_o_-F_c_ density of *ba*_3_ C*c*O from SFX data. (**e**) The F_o_-F_c_ density of *ba*_3_ C*c*O from cryo-LCP data (PDB code 3S8G). (**f**) The F_o_-F_c_ density of bovine heart *aa*_3_-type C*c*O from XFEL data of large crystals at cryo temperature (PDB code 3WG7).

Our SFX structure displays residual electron density for a ligand in the active site. Since this residual density in the unbiased F_o_-F_c_ omit map is spherical in shape it strongly suggests that a single-oxygen species, presumably a water molecule or hydroxide ion, is bound between the heme *a*_3_ iron and Cu_B_ (Figs 3 and 4a). Moreover, the peak height of this active site feature (0.99 e^−^/Å^3^) is very similar to that of a strongly bound water molecule located between Ser309 and the heme *a*_3_ secondary alcohol (0.86 e^−^/Å^3^), further supporting our interpretation that the active site residual density corresponds to a single tightly bound water molecule or hydroxide ion. In contrast, a peroxide molecule was modelled as the active site ligand in the cryo-LCP structure^24^, and the F_o_-F_c_ omit map calculated from the deposited cryo-LCP data clearly displays an elongated electron density peak in the active site (Fig. 4b). When a water molecule is modelled into the active site of the SFX structure, some residual positive F_o_-F_c_ electron density remains (Fig. 4d). However, if a water molecule is modelled into the cryo-LCP structure in place of the peroxide, it results in a poor fit with a much stronger residual density (Fig. 4e). Similarly, the structure of bovine heart *aa*_3_-type C*c*O derived from XFEL diffraction data using large cryo-protected crystals (PDB code 3WG7)^31^ also shows an elongated shape of the active site peak in the F_o_-F_c_ omit map (Fig. 4c). Moreover, if a single water molecule is built into this electron density in place of the peroxide ligand, the fit is poor with a strong residual F_o_-F_c_ density similar to in the cryo-LCP structure (Fig. 4f).

Resonance Raman spectroscopy has shown the existence of a peroxide species bridging the heme *a*_3_ iron and Cu_B_ in the so-called “resting oxidized state” of *aa*_3_-type C*c*O from bovine heart^34^. This has not yet been explicitly investigated for the *ba*_3_ C*c*O, but electrochemically induced UV-vis difference absorbance spectra suggest that there is no peroxide molecule at the active site of *ba*_3_ C*c*O in the oxidized (as prepared) state of the enzyme^35^. On the other hand, it has been demonstrated that the redox sites of *ba*_3_ C*c*O crystals become reduced during X-ray data collection at cryogenic temperatures^33^. Thus the apparent peroxide species in the cryo-LCP structures (Supplementary Table S1) may result from the recombination of two hydroxyl radicals produced from X-ray damage to water molecules near the active site, as the authors also suggest^24^. In contrast, our SFX structure represents a radiation-damage free structure of *ba*_3_ C*c*O for which a water molecule or hydroxide ion is likely the active site ligand.

### Proton translocation pathway in *ba*_3_ C*c*O

The *ba*_3_ C*c*O utilizes a single proton uptake pathway, the so-called K-pathway analogue, in order to provide protons for the oxygen chemistry at the active site, as well as to transport protons that are pumped across the membrane. In the SFX structure we can track a proton pathway that stretches from Glu15^II^ (“II” denotes subunit II), *via* Thr315, Wat721, Tyr248, Thr312, Tyr244, Ser309, Wat738 and the secondary alcohol of heme *a*_3_ to Tyr237 in close proximity of the active site (Fig. 2b). The positions of the two water molecules as well as the side chain conformations of key residues are conserved when comparing with the cryo-LCP structure^24^. Most importantly, the room-temperature SFX structure confirms that there is a gap in the hydrogen bond network between Thr312 and Tyr244, with a distance of 4.45 Å between the two residues. It is possible that transient ordering of a water molecule bridges this gap such that a functional proton pathway is formed when a proton is taken up from the negative side of the membrane.

### Structural differences around the proton-loading site

Transmembrane proton pumping against an electrochemical gradient requires a protonatable group with alternating access to the positive and negative sides of the membrane during the catalytic cycle. There is an ongoing debate as to the identity of this group, referred to as the proton-loading site, where it has been suggested to be localized in the vicinity of the heme *a*_3_ propionates; the surrounding water cluster; or a near-by aspartic acid residue (Asp372 in *ba*_3_ C*c*O)^36, 37^. Several of the residues in this region are conserved throughout the C*c*O superfamily including a tryptophan residue (Trp229 in *ba*_3_ C*c*O). It has been shown that in the *aa*_3_-type C*c*O from *Paracoccus denitrificans* the corresponding residue forms a tryptophan radical during enzymatic turnover^38^. This, however, is not the case for *ba*_3_ C*c*O. It was recently proposed that the transient formation of the tryptophan radical is involved in proton pumping, and consequently the lack of such a radical in *ba*_3_ C*c*O could be related to the lower proton pumping stoichiometry displayed by this enzyme^39^. In light of this debate, it is interesting to note that the SFX structure and all previously published *ba*_3_ C*c*O structures differ from the *aa*_3_-type C*c*O structures in the vicinity of this conserved tryptophan residue (Fig. 2c). Specifically, the tyrosine residue (Tyr136 in *ba*_3_ C*c*O) that hydrogen bonds to the tryptophan is in close proximity of the heme *a*_3_ propionates in *ba*_3_ C*c*O, whereas it is pointing more towards the associated water cluster in the *aa*_3_-type C*c*Os. It may also be of relevance for the difference in proton pumping efficiency that all *aa*_3_-type C*c*Os contain a magnesium ion as part of the above mentioned water cluster. There is no magnesium ion present in our SFX structure or any of the previously deposited *ba*_3_ C*c*O structures. Instead, the position of the magnesium ion is occupied by the carboxyl group of a glutamic acid residue (Glu126^II^) in the *ba*_3_ C*c*O structures (Fig. 2c). Calculations have shown that the magnesium ion affects the proton affinity of the heme *a*_3_ propionate A in the *aa*_3_-type C*c*Os^40^.

## Discussion

We describe a method to prepare large quantities of well-diffracting microcrystals of *ba*_3_ C*c*O in lipidic phase and present the first room-temperature structure of a C*c*O free of radiation damage. The omit electron density map shows that the ligand bound at the active site in the oxidized form of *ba*_3_ C*c*O is most likely a single-oxygen species corresponding to a water molecule or hydroxide ion bound between the heme *a*_3_ iron and Cu_B_. Considering the fact that it is localized between two positively charged groups, the heme *a*_3_ iron and Cu_B,_ a hydroxide ion may be the more likely alternative. Our conclusion is based upon the spherical shape of the electron density peak in the unbiased F_o_-F_c_ omit map and comparison of residual densities when modelling a single water molecule at this site in other deposited C*c*O structures (Fig. 4). It is also apparent that the proton transfer pathway and the water cluster associated with the presumed proton-loading site, described in previous cryo-temperature structures^11, 24^, are conserved at room temperature.

Hirata and co-workers recently presented a structure of bovine heart *aa*_3_-type C*c*O from XFEL diffraction data collected using large cryo-protected crystals where the crystals were physically translated between each X-ray exposure^31^. We propose that the use of microcrystals in order to collect room-temperature SFX data has major advantages over the use of large, cryo-cooled crystals at an XFEL. Specifically, it is clearly beneficial to determine the structure of a protein at room temperature, as this is the more physiologically relevant temperature. Further, each microcrystal is only exposed once to an XFEL pulse when using SFX. This completely avoids the risk of radiation damage accumulating due to X-ray induced free radicals propagating through the crystal. In this study an average X-ray dose of 13 MGy per crystal was used, well below the limit where radiation damage has been detected in metal clusters using XFEL radiation^22^. Microcrystals are also favoured over large crystals if the aim is to trigger a reaction within the crystal. For microcrystals the penetration of the reaction trigger, may it be light or a chemical, is more likely to reach and activate a large population of the protein molecules within the crystal. This idea has been very successfully demonstrated in several recent examples^26, 41, 42^. It is therefore exciting to consider future opportunities for time-resolved structural studies now that we have demonstrated the possibility to collect high resolution SFX data on C*c*O crystals. This opens up a unique opportunity to record structural snapshots of C*c*O as oxygen is reduced to water while protons are simultaneously pumped across a biological membrane.

## Methods

### Expression and purification

Recombinant *ba*_3_ C*c*O from *Thermus thermophilus* was produced and purified as described^43^ with the modifications described below. The cell pellet was resuspended in lysis buffer (50 mM Hepes pH 8.0, 100 mM NaCl, 1 spatula tip of DNase, 2 spatula tips of PMSF) and sonicated. Unbroken cells were collected and sonicated a second time. Membrane proteins were extracted from the membrane twice in extraction buffer (50 mM Hepes pH 8.0, 2.5% Triton X-100); first at 4 °C overnight and then for 4 h at room temperature. Solubilized *ba*_3_ C*c*O was bound to 2 × 5 ml prepacked Ni-NTA columns (HisTrap HP, GE Healthcare Life Science) equilibrated with buffer (10 mM Hepes pH 8.0, 150 mM NaCl, 1% Triton X-100, 10 mM imidazole). The protein was then washed and eluted with equilibration buffer containing 25 mM and 250 mM imidazole, respectively. Pooled fractions were dialyzed against 5 mM Hepes pH 8.0, 0.05% Triton X-100 at 4 °C for 4 h and then against 5 mM Hepes pH 8.0, 0.05% dodecyl-β-D-maltoside at 4 °C overnight. The purified protein was stored in glass vials at 4 °C at a concentration of 25–30 mg/ml.

### Crystallization

Purified *ba*_3_ C*c*O was reconstituted in a lipidic cubic phase (LCP) and microcrystals for serial femtosecond crystallography were produced by optimizing previous crystallization conditions^24^ following a reportedly successful workflow^25^. Crystallizations were performed in 9-well glass plates where a string of protein-lipid mixture was added to 300 μl precipitant solution and covered by a sheet of plastics, or in Hamilton glass syringes. Crystals of 5–20 μm in size were obtained within 2–3 days at 19 °C in 37% (v/v) PEG 400, 1.0–1.4 M NaCl, 100 mM sodium cacodylate trihydrate pH 5.3 and stored in Hamilton gastight syringes.

### Absorbance spectroscopy

Microcrystals of *ba*_3_ C*c*O were prepared as described above. 10 μl crystal-containing LCP was added to a CaF_2_ glass cell with a path length of 0.2 mm and the absorbance spectrum recorded between 200 and 700 nm in a Hitachi U-2910 spectrophotometer. The absorption spectrum of the monoolein mixed with buffer only was subtracted from the spectrum of the samples. To record a spectrum of the reduced sample, sodium dithionite was mixed with monoolein that was subsequently added to the crystal-containing LCP, giving a final dithionite concentration of 4.8 mM. The final protein concentration in the LCP was approximately 0.1 mM for both samples. However, the local crystal concentration in the cell may vary between different measurements.

### Data collection and processing

15 μl LCP-crystals of *ba*_3_ C*c*O were homogenized with 45 μl monoolein (Nu-Check Prep Ink. CAS: 111–03–5) prior to loading into the LCP injector (described in detail in ref. 26). SFX diffraction data was collected at the BL3 beamline at the Spring-8 Angstrom Compact Free Electron Laser (SACLA)^44^ at 7.6 keV with a repetition rate of 30 Hz and a pulse duration of <10 fs. The X-ray pulses were focused to 1.5 μm (height) ×1.3 μm (width) by Kirkpatrick-Baez mirrors, where the pulse energy at the sample position was estimated at approximately 95 μJ per pulse. The LCP-crystals were injected into the X-ray beam through a 75 μm diameter nozzle at a flow rate of 0.48 μl/min. The sample-to-detector distance was 53 mm and data was recorded using an eight sensor module CCD detector^45^. Diffraction images were processed using the software package Cheetah^27^ through a pipeline adapted for SACLA^46^. Images containing more than 20 diffraction peaks were identified as hits and further processed with CrystFEL^28^ where DirAx^47^ and Mosflm^48^ were used for indexing. Intensities from all images were merged and scaled using Partialator without partiality correction. A per-crystal resolution cut-off was employed that rejected data beyond 1.2 Å past the crystal resolution as calculated by the peak finding in CrystFEL. The crystal hit rate was about 12% and the indexing rate was 72%. The average radiation dose per crystal was calculated using the program RADDOSE-3D^49^. This can be considered as an upper estimate of the actual dose due to the possibility that photoelectrons escape from the microcrystals, which is not taken into account in the calculation.

### Structure determination and refinement

The structure was solved by molecular replacement with Phaser^29^ using the high-resolution cryo-LCP wild type *ba*_3_ C*c*O structure (PDB code 3S8F) as a template. The model was built in Coot^50^ and refinement at a resolution of 2.3 Å was carried out in REFMAC5^51^ using anisotropic displacements with three rigid-body motion groups and an X-ray weighting term of 0.05. Final *R*_work_ and *R*_free_ were 16.2% and 19.8% respectively with 99.6% of the side chains within accepted Ramachandran regions. Data collection and refinement statistics are summarized in Table 1.

**Table 1:** Data collection and refinement statistics.

PDB Code	5NDC
**Data Collection**	
Collection temperature (K)	293
Space Group	C121
Cell dimensions	
a, b, c (Å)	145.9, 100.3, 96.6
α, β, γ (°)	90, 126.8, 90
Resolution (Å)^‡^	36.4–2.3 (2.34–2.30)
R_split_ (%)^†,‡^	19.37 (120)
I/σ(I)^‡^	3.74 (1.02)
CC(1/2)^‡^	95.6 (36.6)
Completeness (%)	100
Multiplicity^‡^	36.8 (14.7)
Number of collected images	87057
Number of hits	11374
Number of indexed patterns	8211
Indexing rate (%)	72.2
Number of total reflections	1864107
Number of unique reflections	50602
**Refinement**	
Resolution	36.4–2.3
R_work_/R_free_ (%)	16.2/19.8
Number of atoms	6386
Average B factor (Å^2^)	43.9
R.m.s deviations	
Bond lengths (Å)	0.012
Bond angles (°)	1.61

^†^ $${{\rm{R}}}_{{\rm{split}}}=1/\sqrt{2\,\frac{{\sum }^{}hkl|{\boldsymbol{I}}even-{\boldsymbol{I}}odd|}{1/2{\sum }^{}hkl|{\boldsymbol{I}}even+{\boldsymbol{I}}odd|}}$$.

^‡^Values in parenthesis is those of the highest resolution shell.

## Electronic supplementary material

Supplementary Information
