# Supplementary material for: Serial femtosecond crystallography structure of cytochrome c oxidase at room temperature
Source: Sci Rep. 2017 Jul 3;7:4518. doi: 10.1038/s41598-017-04817-z (PMC5495810; doi:10.1038/s41598-017-04817-z)
Supplement: Supplementary file 1 — Supplementary Information [file 41598_2017_4817_MOESM1_ESM.pdf]

Supplementary Information

Serial femtosecond crystallography structure of cytochrome *c* oxidase at room temperature

Rebecka Andersson[1], Cecilia Safari[1], Robert Dods[1], Eriko Nango[2,3], Rie Tanaka[2], Ayumi Yamashita[2], Takanori Nakane[4], Kensuke Tono[5], Yasumasa Joti[5], Petra Båth[1], Elin Dunevall[1], Robert Bosman[1], Osamu Nureki[4], So Iwata[2,3], Richard Neutze[1] & Gisela Brändén[1*]

[1]Department of Chemistry and Molecular Biology, University of Gothenburg, Box 462, SE-40530 Gothenburg, Sweden.

[2]RIKEN Spring-8 Center, 1-1-1 Kouto, Sayo-cho, Sayo-gun, Hyogo 679-5148, Japan.

[3]Department of Cell Biology, Graduate School of Medicine, Kyoto University, Yoshidakonoe-cho, Sakyo-ku, Kyoto 606-8501, Japan.

[4]Department of Biological Sciences, Graduate School of Science, University of Tokyo, 2-11-16 Yayoi, Bunkyo-ku, Tokyo 113-0032, Japan.

[5]Japan Synchrotron Radiation Research Institute, 1-1-1 Kouto, Sayo-cho, Sayo-gun, Hyogo 679-5198, Japan

[*]To whom correspondence should be addressed: gisela.branden@gu.se

Supplementary Figure S1.

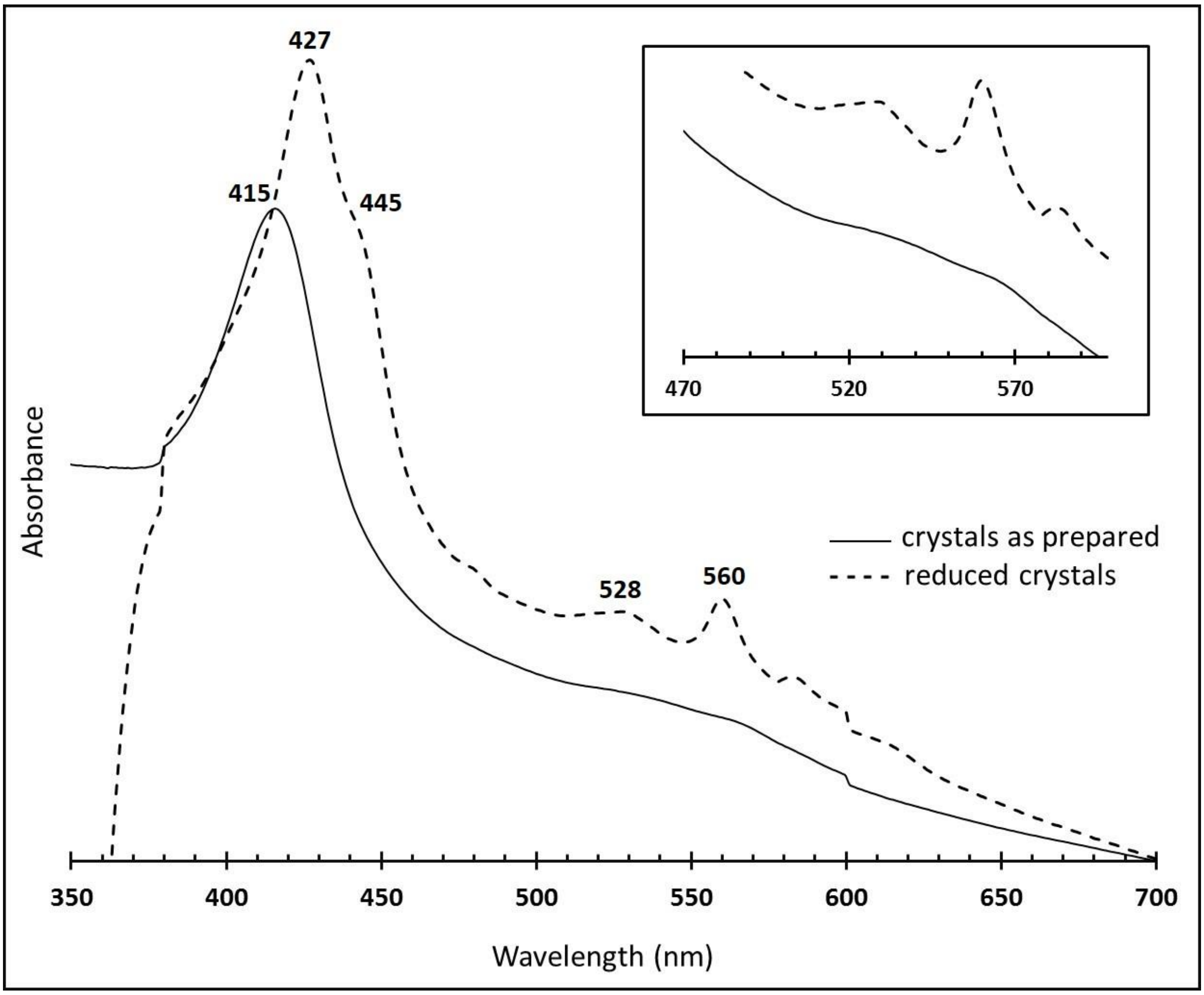

Supplementary Figure S1. Optical absorbance spectra of *ba*3 C*c*O microcrystals. The spectra for microcrystals as prepared (oxidized state) and after addition of dithionite (reduced state) are shown. The oxidized sample has a peak at 415 nm and the reduced sample at 427 nm, 445 nm (shoulder), 528 nm and 560 nm.

Supplementary Figure S2.

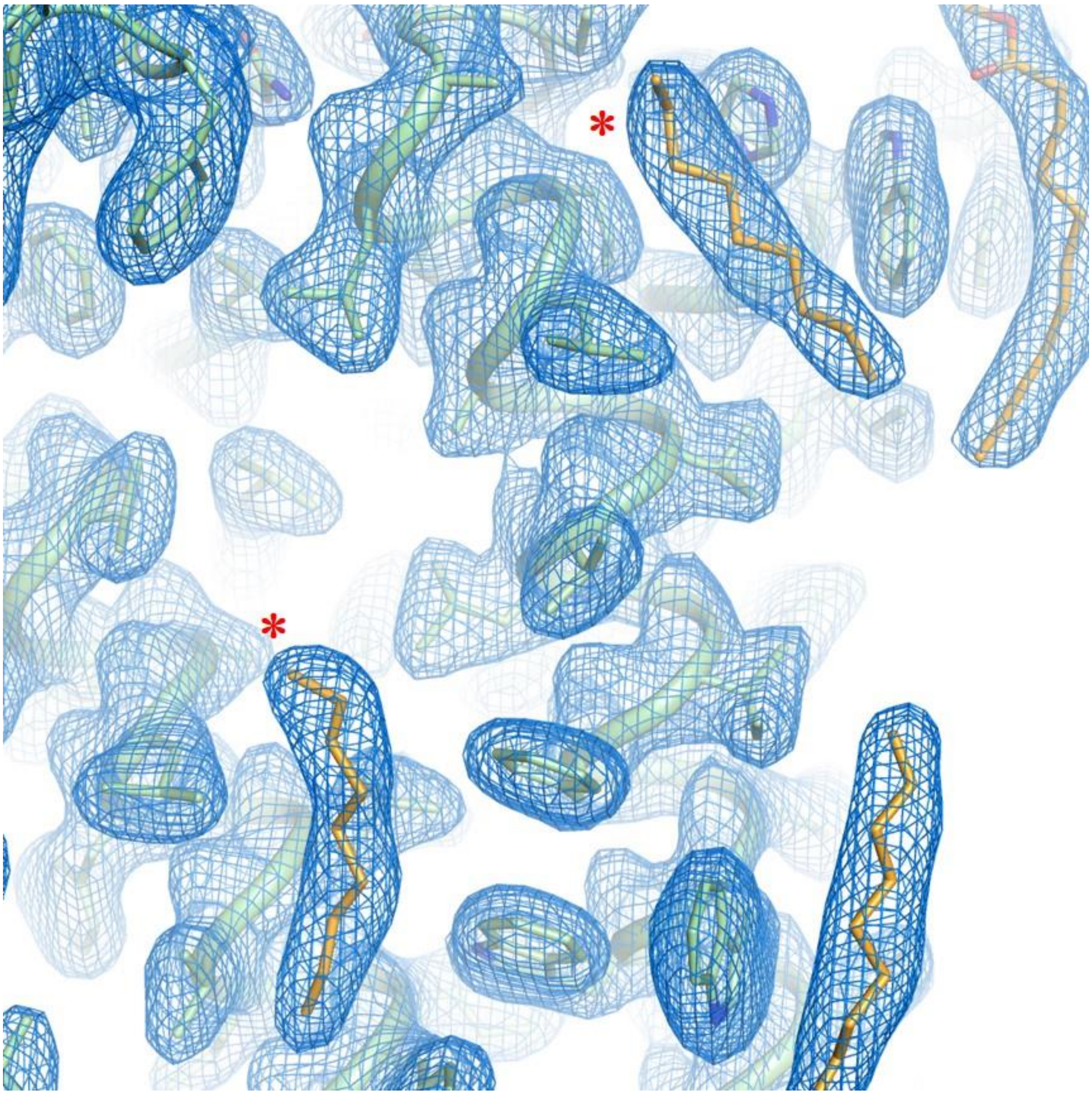

Supplementary Figure S2. SFX structure of *ba*$_3$ C*c*O displaying previously unresolved lipid molecules. Subunit I is shown in green and lipid molecules in orange. The $2F_o$-F_c density (blue) is contoured at 1.0 σ. Newly resolved lipid tails are highlighted with an asterisk.

Supplementary Figure S3.

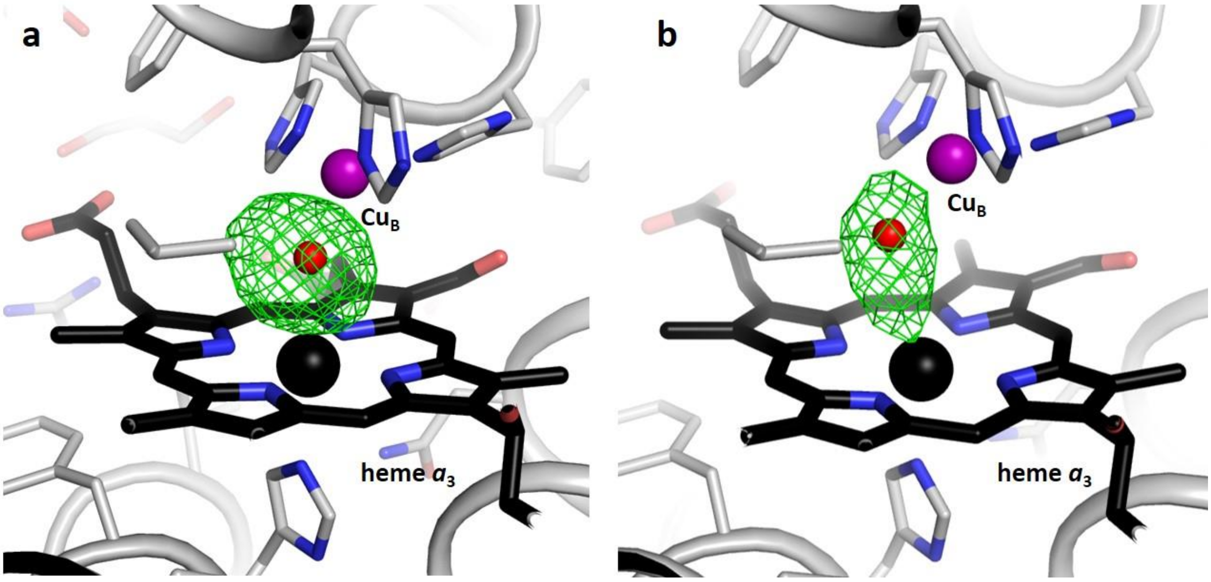

Supplementary Figure S3. Active site electron densities in *ba*3 C*c*O structures crystallized by vapor diffusion. F_o-F_c omit map densities (green) contoured at 4.5 σ, calculated without any active site ligand for (**a**) PDB code 1XME and (**b**) PDB code 3EH4. For all structures resulting from vapor diffusion crystallization, a water molecule or no ligand is modeled in the active site. In contrast, elongated active-site electron density in the deposited LCP structures is interpreted as a peroxide ligand (Supplementary Table S1).

Supplementary Table S1.

PDB code	year released	crystallization method	resolution (Å)	active-site ligand	comment
1EHK	2000	vapor diffusion	2.4	water	no structure factors deposited
1XME	2005	vapor diffusion	2.3	water	
2QPE	2007	vapor diffusion	2.9	none	two surface mutations
2QPD	2007	vapor diffusion	3.25	none	one surface mutation
3EH5	2009	vapor diffusion	2.8	none	reduced enzyme
3EH4	2009	vapor diffusion	2.9	water	crystals reduced in X-ray beam
3EH3	2009	vapor diffusion	3.1	none	crystals reduced by dithionite
3S8G	2011	LCP	1.8	peroxide	
3S8F	2011	LCP	1.8	peroxide	
3QJV	2012	vapor diffusion	2.8	CO	CO-pressurized crystals
3QJU	2012	vapor diffusion	2.9	CO	CO-pressurized crystals
3QJT	2012	vapor diffusion	2.95	CO	CO-pressurized crystals
3QJS	2012	vapor diffusion	2.8	CO	CO-pressurized crystals
3QJR	2012	vapor diffusion	3.2	CO	CO-pressurized crystals
3QJQ	2012	vapor diffusion	2.9	CO	CO-pressurized crystals
3S3D	2012	vapor diffusion	3.75	none	Xe-pressurized crystals
3S3C	2012	vapor diffusion	4.0	none	Xe-pressurized crystals
3S3B	2012	vapor diffusion	3.3	none	Xe-pressurized crystals
3S3A	2012	vapor diffusion	4.25	none	Xe-pressurized crystals
3S39	2012	vapor diffusion	4.8	none	Xe-pressurized crystals
3S38	2012	vapor diffusion	4.2	none	Xe-pressurized crystals
3S33	2012	vapor diffusion	4.45	none	Xe-pressurized crystals
4FA7	2012	LCP	2.5	peroxide	A204F mutation
4FAA	2012	LCP	2.8	peroxide	A120F+A204F mutations
4GP8	2013	LCP	2.8	peroxide	Y133W+T231F mutations
4GP5	2013	LCP	2.7	peroxide	Y133W mutation
4GP4	2013	LCP	2.8	peroxide	Y133F mutation
4G7S	2013	LCP	2.0	peroxide	V236I mutation
4G7R	2013	LCP	3.05	peroxide	V236A mutation
4G7Q	2013	LCP	2.6	peroxide	V236L mutation
4G72	2013	LCP	3.19	peroxide	V236M mutation
4G71	2013	LCP	2.9	peroxide	V236N mutation
4G70	2013	LCP	2.6	peroxide	V236T mutation
4N4Y	2014	LCP	2.9	peroxide	G232V mutation

Supplementary Table S1. A summary of *ba*$_3$ C*c*O structures deposited in the PDB.
